# Vestibular Facilitation of Optic Flow Parsing

**DOI:** 10.1371/journal.pone.0040264

**Published:** 2012-07-02

**Authors:** Paul R. MacNeilage, Zhou Zhang, Gregory C. DeAngelis, Dora E. Angelaki

**Affiliations:** 1 Vertigo, Balance, and Oculomotor Research Center, University Hospital of Munich, Munich, Germany; 2 Department of Biomedical Engineering, University of Southern California, Los Angeles, California, United States of America; 3 Department of Brain and Cognitive Sciences, University of Rochester, Rochester, New York, United States of America; 4 Department of Neuroscience, Baylor College of Medicine, Houston, Texas, United States of America; University of Muenster, Germany

## Abstract

Simultaneous object motion and self-motion give rise to complex patterns of retinal image motion. In order to estimate object motion accurately, the brain must parse this complex retinal motion into self-motion and object motion components. Although this computational problem can be solved, in principle, through purely visual mechanisms, extra-retinal information that arises from the vestibular system during self-motion may also play an important role. Here we investigate whether combining vestibular and visual self-motion information improves the precision of object motion estimates. Subjects were asked to discriminate the direction of object motion in the presence of simultaneous self-motion, depicted either by visual cues alone (i.e. optic flow) or by combined visual/vestibular stimuli. We report a small but significant improvement in object motion discrimination thresholds with the addition of vestibular cues. This improvement was greatest for eccentric heading directions and negligible for forward movement, a finding that could reflect increased relative reliability of vestibular versus visual cues for eccentric heading directions. Overall, these results are consistent with the hypothesis that vestibular inputs can help parse retinal image motion into self-motion and object motion components.

## Introduction

Accurate and precise estimation of object motion during self-motion is important for survival, because moving organisms must often simultaneously monitor other moving agents, including predators, prey and potential mates. Self-motion relative to a stationary environment produces a globally consistent pattern of visual motion on the retina, whereas independently moving objects give rise to local motion signals that are inconsistent with the global pattern. Thus, estimating object motion during self-motion can potentially be achieved by comparing local retinal motion signals to the global flow pattern. Indeed, visual psychophysical studies in humans have shown that the brain parses retinal image motion into object and self-motion components based on global flow computations [1]–[9]. This body of research has focused on two related topics: 1) estimating heading (i.e., direction of self-translation) in the presence of moving objects [1], [3], [10], [11], and 2) estimating object motion during self-motion [2], [4]–[9], [12], [13].

These studies, however, have primarily focused on biases introduced by interactions between object motion and background motion due to self-translation, and have not generally considered how these interactions affect perceptual sensitivity. Furthermore, while some prior studies have investigated perception of object motion during real physical self-motion [14], [15], other studies that have focused on the specific question of optic flow parsing have largely ignored non-visual (e.g., vestibular and proprioceptive) cues that could help to disambiguate retinal image motion. In particular, vestibular sensory signals play a vital role in heading perception, leading to more precise heading estimates when both visual and vestibular cues are available [16]–[19]. Given these interactions between self-motion and object motion perception, as documented previously, we hypothesized that vestibular signals may also influence the precision with which subjects judge object motion during self-motion.

To test this hypothesis, we asked subjects to discriminate object motion during simulated self-motion in the presence and absence of scene-consistent vestibular stimulation. Our rationale is as follows: combined visual/vestibular stimulation leads to improved heading perception [16]–[19] and thus presumably improved flow estimation at the object location, and may therefore also lead to improved flow parsing ability and object motion discrimination. The vestibular contribution to heading perception depends on the relative reliability of visual and vestibular cues, so we hypothesized that the same should hold for flow-parsing and object motion discrimination. Relative reliability was manipulated by varying heading eccentricity (i.e., heading direction relative to straight ahead). Relative reliability of vestibular cues increases with eccentricity because visual heading discrimination thresholds increase more steeply with eccentricity than vestibular thresholds [20], [21]. Therefore we expected that improvement in object motion discrimination thresholds during the combined visual-vestibular stimulation would be more pronounced for eccentric rather than forward heading directions. Preliminary aspects of this work were presented in abstract form [22], [23].

## Methods

### Ethics Statement

Eight human subjects (3 female) participated in this study. Informed consent was obtained from all participants and all procedures were reviewed and approved by the human subjects committee of Washington University.

### Setup

Subjects were seated in a padded racing seat mounted on a 6-degree-of-freedom Moog© motion platform. A 3-chip DLP projector (Galaxy 6; Barco, Kortrijk, Belgium) was also mounted on the motion platform behind the subject and front-projected images onto a large (149×127 cm) projection screen via a mirror mounted above the subject’s head. The projection screen was located ∼70 cm in front of the eyes, thus allowing for a visual angle of ∼94°×84°. A 5-point harness held subjects’ bodies securely in place and a custom-fitted plastic mask secured the head against a cushioned head mount thereby holding head position fixed relative to the chair. Subjects were enclosed in a black aluminum superstructure, such that only the display screen was visible in the darkened room. Subjects also wore active stereo shutter glasses (CrystalEyes 3; RealD, Beverly Hills, CA), thereby restricting the field of view to ∼90°×70°. Eye position was recorded for both eyes at 600 Hz via a video-based eye-tracking system (ISCAN©) attached to the stereo glasses and subjects were instructed to look at a centrally-located, head-fixed target throughout each trial. Sounds from the platform were masked by playing white noise through headphones. Behavioral tasks and data acquisition were controlled by Matlab and responses were collected using a button box. Additional details specific to the human apparatus can be found in recent publications [18], [21], [24].

### Experimental Protocol: Main Experiment

The visual scene consisted of a 3-dimensional (3D) starfield composed of randomly placed triangles with base and height of 1 cm. The triangles filled a volume 170 cm wide ×170 cm tall× 100 cm deep and the 3D density of triangles was 0.001 triangles/cm^3^. With this density and viewing frustum, ∼1000 triangles were rendered on a given frame. The nearest and farthest rendered triangles subtended ∼3° and ∼0.6°, respectively. A spherical object (diameter of 10 cm, i.e., ∼8°) was rendered at the same depth as the screen, and located to the left of the fixation point, ∼27 cm (∼21°) away. The object was also composed of random triangles and the density of triangles within the volume of the object was the same as for the starfield, such that the object was distinguished only by its velocity relative to the background motion. Given the volume of the sphere and its density, ∼4 triangles were rendered within the sphere on a given video frame. Motion coherence of the starfield and object was set to 70% and the elements of the scene were limited-lifetime (1 sec). Note, reduced motion coherence was used to make the relative reliabilities of the visual and vestibular self-motion cues more equal [17], [18], and to allow comparison with heading discrimination data collected under the same conditions with a range of heading eccentricities [21]. To prevent pop-out of the object relative to the background, object motion coherence matched coherence of the background star field.

**Figure 1:**
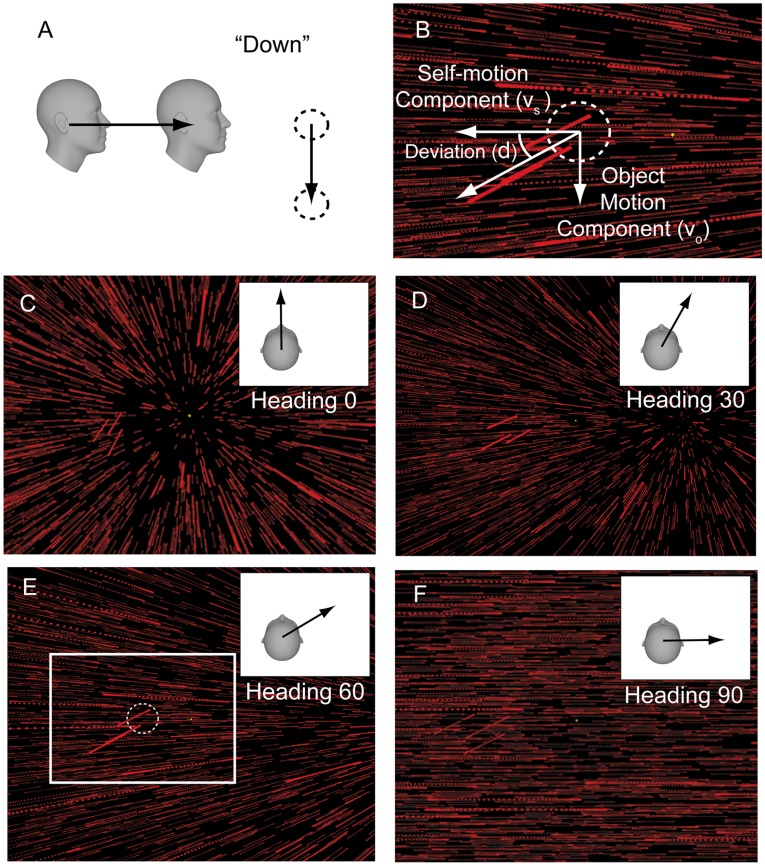
Schematic of the experimental design. A) Side-view illustrating the task with a heading of 0° (straight forward). The subject experiences self-motion and synchronized movement of the object (dashed circle) either up or down. The subject’s task is to indicate which direction the object moved in the world. B) Close up of the pattern of image motion on the display for heading  = 60° and downward object motion in the world (from panel E). Variables *v_s_* and *v_o_* represent the independent components of image motion associated with the self-motion and object motion, respectively (horizontal and vertical white arrows). Note that the object motion component (*v_o_*) is equal in all examples shown here (C-F), but the angle of deviation (*d*) is not because the self-motion component (*v_s_*) depends on heading direction. (C)-(F) The experiment was conducted at four heading directions: 0°, 30°, 60°, and 90°. The optic flow associated with each heading direction (as displayed on the screen) is illustrated in each panel and each inset shows a top down view of the self-motion trajectory. As heading eccentricity increases, the focus of expansion (FOE) is displaced further from the center of the display. The resultant image motion associated with the object is also visible in these panels to the left of fixation.

Each trial simulated a 13cm, 1s translation of the subject relative to the starfield and object. The object was simultaneously displaced either upward or downward relative to the starfield and the subject’s task was to indicate whether the object moved upward or downward relative to the world (Fig. 1A). Note that we did not attempt to evaluate whether subjects made their judgments in world or screen coordinates. However, regardless of the coordinate frame of the judgment, subjects had to parse the optic flow field to perform the task. Thus, for this task, we do not suspect that the basic conclusions of the present study would change depending on the strategy used by the subjects.

The simulated self-motion and object motion followed synchronized Gaussian velocity profiles, such that the object could not be distinguished simply by having a different temporal profile of motion than the background. Given this velocity profile, the peak simulated visual and vestibular speed of self-motion was 30 cm/s and peak acceleration/deceleration was 1.13 m/s^2^. This dynamic stimulus was chosen because: (1) it is a smooth, transient, natural stimulus, (2) it evokes robust visual and vestibular responses in cortical multisensory neurons (e.g., areas MSTd and VIP; both visual and vestibular responses tend to reflect stimulus velocity more than acceleration [25]–[28]), (3) it results in near-optimal multisensory integration, both at the level of behavior [17]–[19] and at the level of single neurons [17], [19], [29].

Due to the independent object motion in the scene, the retinal image motion associated with the object deviated from that of the surrounding optic flow (Fig. 1B). Deviation angle was varied from trial to trial according to a staircase procedure. The staircase began at the largest deviation angle and possible deviation angles were +/− [80° 64° 48° 32° 16° 8° 4° 2° 1° 0.5° 0.25°]. The deviation angle was reduced 30% of the time after correct responses and was increased 80% of the time after incorrect responses. This staircase rule converges to the 73% point of the psychometric function. The deviation angle was positive (upward) on 50% of trials and negative (downward) on the other 50%.

The angle of deviation is given by 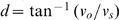 where *v_s_* and *v_o_*, respectively, are the independent velocity components (in screen coordinates) associated with self-motion and object motion, respectively (Fig. 1B). The self-motion component (*v_s_*) depended on heading angle but was constant for a given heading (peak velocity of 10.2°/s, 20.7°/s, 24.0°/s, and 20.8°/s for headings of 0°, 30°, 60°, and 90°, respectively). Deviation angle (d) for a given trial was specified by the staircase procedure. Object speed on the screen (*v_o_*) was therefore constrained to satisfy the above equation.

Four different heading directions were examined (0°, 30°, 60°, and 90° from straight ahead, Fig. 1C-F), with data for each heading angle collected in a separate block of trials. Trials for visual-only and combined (visual/vestibular) conditions were interleaved within a given block (200 trials/block, lasting ∼25 min). This made for a total of 8 stimulus conditions in the *Main Experiment*. At least 800 trials per condition per subject (6 subjects, S1-S6) were collected.

### Experimental Protocol: Eye-movement Control

Because no eye movement data were recorded initially, we repeated the visual-only and combined protocols in a second experiment for the lateral (90°) heading only, while recording eye movements. This was necessary to verify that subjects maintained fixation equally well during both visual-only and combined visual-vestibular trials. At least 500 trials per subject per condition were collected in 5 subjects (S4-S8) for the second experiment.

### Experimental Protocol: Retinal-speed Control

Finally, in a third experiment, observers were presented with visual-only trials, as described above, except that the simulated distance of translation was reduced to <13cm (6.75, 5.56, and 6.13 cm for heading directions of 30°, 60° and 90°, respectively) in order to achieve the same retinal image speed (*v_s_* in Fig. 1B) at the eccentric location where the moving object was presented (*v_s_* equal to 10.2°/s for all headings). This control experiment was necessary to examine to what extent the observed dependence of object motion discrimination thresholds on heading direction was simply a result of changes in retinal speed. Because translation distance was fixed in the first experiment, *v_s_* increases with eccentricity, such that effects of heading eccentricity (i.e. flow-field geometry) and retinal speed are confounded. At least 600 trials per subject per condition were collected in 5 subjects (S4-S8) for the third experiment.

### Data Analysis

For each subject and each condition we plotted the proportion of ‘upward’ responses as a function of object deviation angle and a cumulative Gaussian function was fit to these data using psignifit software [30], [31]. Threshold is given by the standard deviation of the fitted function. A two-factor repeated measures ANOVA was performed on threshold data from the Main Experiment to examine the effect of heading eccentricity (0°, 30°, 60°, 90°), the effect of condition (visual-only, combined), and their interaction. Data were further examined using paired t-tests. Threshold data from the Retinal-speed Control experiment were analyzed with a one-factor repeated measures ANOVA to examine the effect of heading eccentricity (0°, 30°, 60°, 90°) when retinal speed at the object location was matched across headings.

To analyze eye movement data, horizontal eye position traces were first smoothed by applying a boxcar filter and then differentiated to obtain eye velocity traces for both eyes. From these traces we calculated mean eye velocity during the stimulus presentation (1s) on each trial and then examined how psychophysical threshold changed as a function of mean eye velocity for each subject. Over the entire range of mean eye velocities, we used a sliding window 1°/s wide, and fit a psychometric function to all trials within that window, provided that a minimum of 150 trials were available in a given velocity window. Window position was increased from the minimum to the maximum mean velocity at 0.1°/s intervals, so that a different threshold was calculated for each window position (i.e., each mean eye velocity). A regression line was fit to the resulting data and the slope and significance of the regression were used to evaluate the influence of mean eye velocity on discrimination performance.

## Results

In these experiments, optic flow simulated observer translation through a starfield, while simultaneously an object moved up or down in the world (Fig. 1A). The subject’s task was to indicate the object’s motion direction (up/down) in the world during trials in which self-motion was cued by either optic flow alone (visual-only condition) or optic flow combined with platform motion (combined condition). The object was transparent, composed of random dots with the same density as the starfield, and was distinguished from the starfield only by the relative velocity of its movement. Starfield and object velocity followed synchronized Gaussian velocity profiles. Object motion amplitude (i.e., total displacement), and thus angle of deviation of the object motion relative to the background (Fig. 1B), was varied from trial to trial using a staircase procedure. Subjects were instructed to maintain visual fixation on a central, head-fixed target to cancel reflexive eye movements. In each block of trials, the heading was fixed, but it differed across blocks such that data were collected separately for forward (0°), lateral (rightward, 90°) and intermediate (30° and 60°) directions (Fig. 1).

### Main Experiment

**Figure 2:**
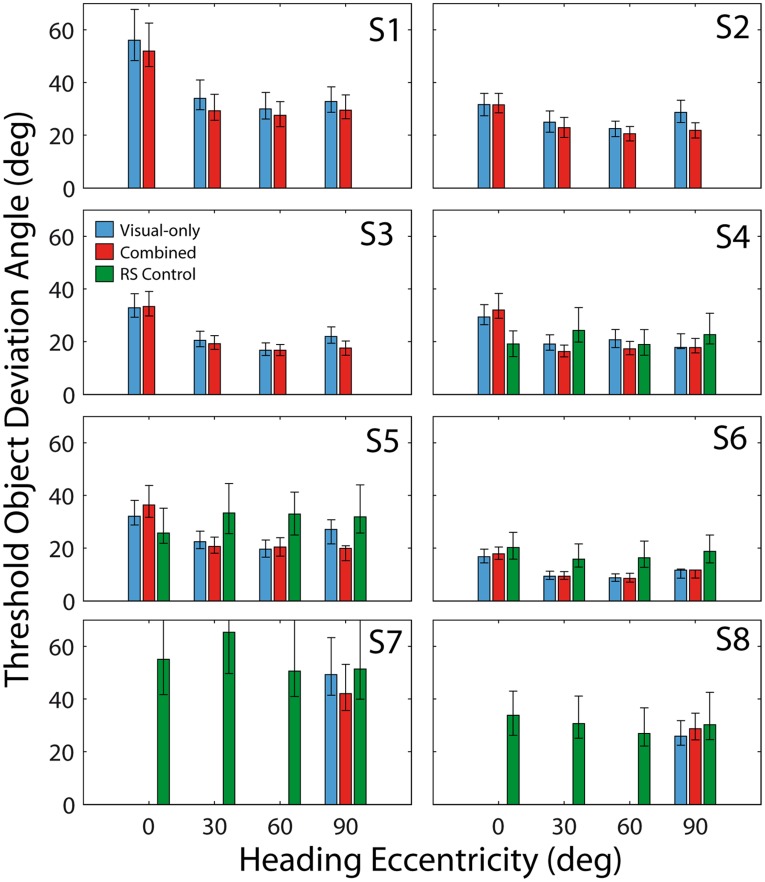
Summary of discrimination thresholds. Each panel shows the data from a different subject. Error bars represent 95% confidence intervals. Subjects S1-S6 participated in the main experiment, so visual-only (blue bars) and combined (red bars) thresholds were measured at all heading eccentricities. Subjects S4-S8 participated in the retinal speed (RS) control experiment (green bars). Note that subjects S7 and S8 were only tested with the 90° heading in the eye movement control experiment (lateral motion).

Subject-by-subject thresholds for both the visual-only and combined conditions are displayed in Fig. 2 (blue and red bars, respectively). For most subjects and most headings, it can be observed that combined thresholds are slightly lower than those in the visual-only condition; this effect was significant. Across all heading eccentricities, the mean object discrimination threshold is lower in the combined condition compared to the visual-only condition (p = 0.011; paired t-test), consistent with the hypothesis that vestibular cues facilitate optic flow parsing. A separate analysis also revealed a significant effect of stimulus condition on threshold improvement (combined vs. visual-only: F(1,5) = 7.40, p = 0.04, repeated measures ANOVA).

**Figure 3:**
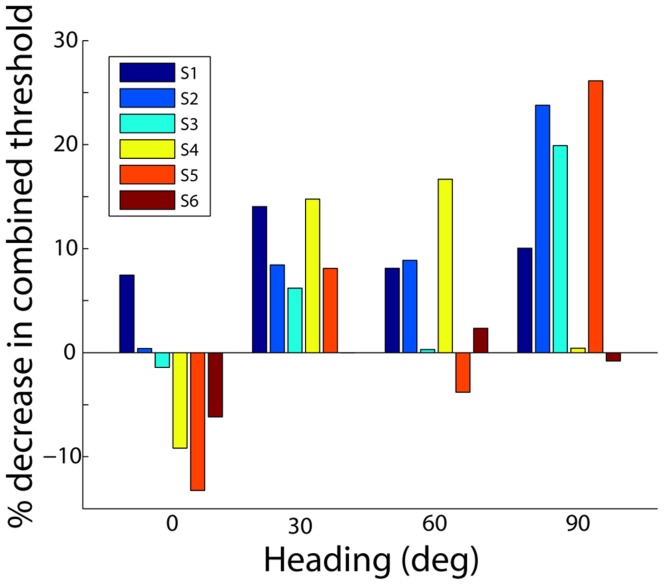
Comparison of visual-only and combined thresholds. Percent decrease in combined threshold relative to the visual-only threshold (computed as *(* ***σ*** *_v_ -* ***σ*** *_c_)/* ***σ*** *_v_*; subjects S1-S6) for all four heading angles. The decrease in threshold depends on heading angle, with the smallest decrease for 0° heading and the largest decrease for 90° heading.

Closer examination of Fig. 2 reveals that the improvement in object discrimination thresholds in the combined condition depends on heading eccentricity, and this effect was also significant (F(3,5) = 3.78, p = 0.03, interaction term of repeated measures ANOVA). This dependence of vestibular facilitation on heading eccentricity is further illustrated in Fig. 3, which plots the percentage decrease in object discrimination thresholds in the combined condition, relative to that in the visual-only condition, for subjects that participated in all conditions of the main experiment (S1-S6). For the forward (0°) heading, there was no significant improvement in object discrimination thresholds when vestibular cues were present (p = 0.58; paired t-test). In contrast, for headings 30°, 60°, and 90°, the improvement was either significant or approaching significance (p = 0.02, p = 0.12, p = 0.04, respectively; paired t-test). Pooling across all non-zero heading directions, the improvement was highly significant (p<0.001; paired t-test).

As shown in Fig. 3, vestibular facilitation was least for heading 0 deg, greatest for heading 90 deg, and moderate for intermediate heading angles. The corresponding mean percentage decreases in the combined condition were −3.1%, 9.7%, 6.7%, and 17.0% for headings 0°, 30°, 60°, and 90°, respectively. While we do not expect vestibular facilitation to depend linearly on heading eccentricity, the data suggests a trend for vestibular facilitation to increase with heading eccentricity. Therefore, using the data presented in Fig. 3, we conducted a non-parametric (rank-based) correlation analysis in order to evaluate the significance of this trend. This revealed a significant positive correlation between heading eccentricity and percent decrease in combined threshold (p = 0.007, Spearman’s rho  = 0.53).

### Eye-movement Control

A potentially trivial explanation for this finding is that incomplete suppression of the translational vestibulo-ocular reflex (TVOR) improves nulling of retinal slip in the combined condition compared to the visual-only condition. In this scenario, a residual TVOR during combined stimulation would *physically* (rather than *computationally* through flow parsing) cancel more of the background motion on the retina, thus reducing the speed of the starfield motion and making it easier to discriminate the direction of object motion. Indeed, prior research has shown that the TVOR is more effective in canceling retinal slip during lateral than during forward movements [32]–[34], consistent with the improvement we observed during lateral self-motion. We therefore repeated the experiment for the lateral (90°) heading in a subset of subjects (S4-S8) while recording eye movements, in order to monitor fixation and identify differences in residual eye velocity between visual-only and combined conditions.

**Figure 4:**
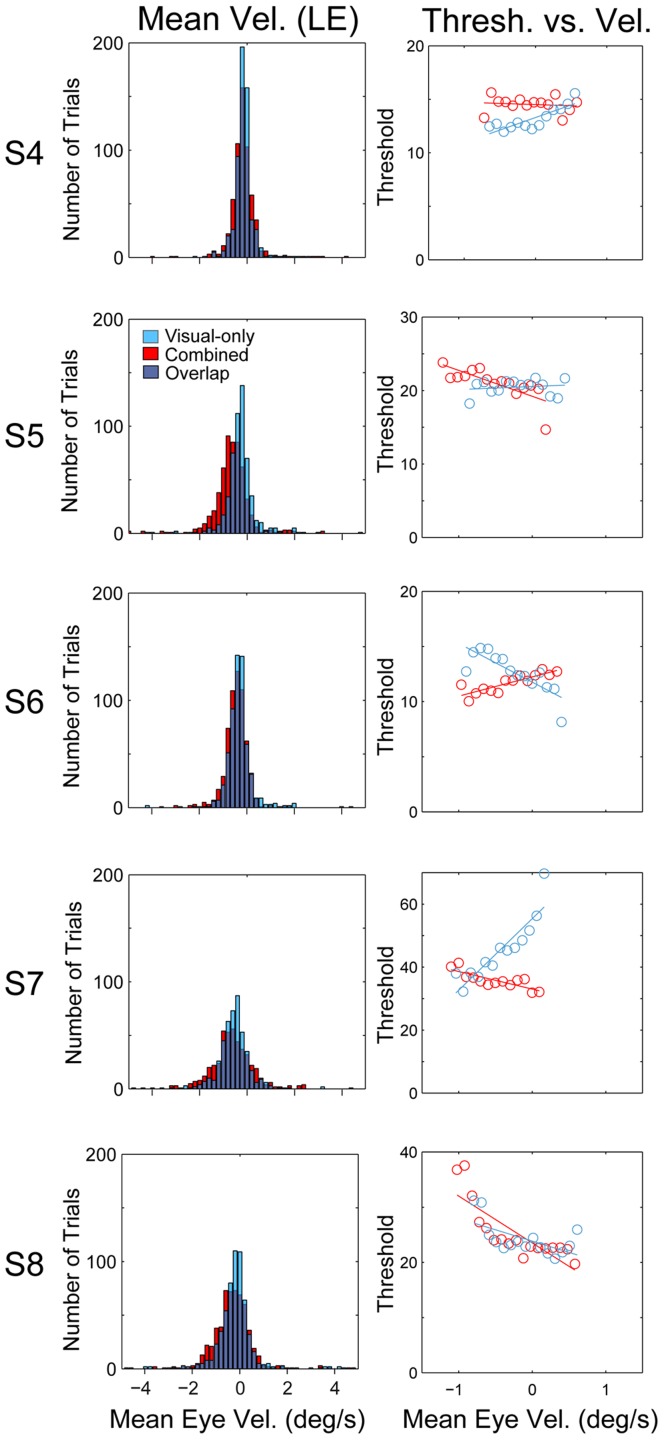
Summary of eye movement analysis. Each row summarizes data from one subject. Only left eye (LE) velocities were used for these analyses; conducting the same analyses using right eye velocities yielded similar results. Left column shows histograms of mean eye velocities from all trials for both the Visual-only (blue) and Combined (red) conditions. Right column shows Visual-only (blue) and Combined (red) thresholds as a function of mean eye velocity, along with regression lines fit to these data (see text for details).

Distributions of mean eye velocity (for the left eye) are illustrated in Fig. 4, left column (blue: visual-only condition; red: combined condition). Because the self-motion direction was rightward in these experiments, an unsuppressed TVOR would elicit leftward (negative) eye velocities. All histograms peaked near zero with only one subject (S6) exhibiting mean eye velocity significantly different from zero (t-test, visual-only p<0.001, combined p = 0.01). Importantly, visual-only and combined histograms were largely overlapping; there was no significant difference in the distribution of eye velocity between combined and visual-only conditions, and this was true for all subjects (t-test, p>0.05). To further investigate the relationship between eye movements and object discrimination performance, we also examined how object discrimination thresholds changed as a function of mean eye velocity for each subject. To do this, we binned trials according to mean eye velocity and we fitted psychometric functions to behavioral data for each bin (see Methods for details). If a residual TVOR facilitates object motion discrimination in the combined condition (red), there should be a positive correlation between mean eye velocity and discrimination performance (i.e., leftward (negative) eye velocity should be associated with lower thresholds).

Only one subject (S6) exhibited a significant positive correlation between eye velocity and discrimination threshold in the combined condition (r = 0.85, p<0.001). However, visual-only and combined thresholds were virtually identical for this subject (Fig. 2, S6, Heading = 90°). On the other hand, subjects who exhibited the largest decrease in threshold for the combined relative to the visual-only condition (e.g. S5 or S7) showed a negative correlation for the combined condition in Fig. 4 (larger leftward eye velocities were associated with *worse* discrimination performance; S5, r = −0.76, p = 0.001; S7, r = −0.82, p<0.001). Moreover, S7 showed a significant positive correlation between threshold and eye velocity for the visual-only condition (r = 0.90, p<0.001), suggesting that unsuppressed (perhaps optokinetic) eye movements led to improved performance in the visual-only but not in the combined condition. Yet this subject performed better in the combined that the visual-only condition, suggesting that these correlations cannot explain the behavioral results. Thus, in summary, we found no evidence that the improvement in object discrimination thresholds in the combined condition is due to a physical cancellation of the optic flow by unsuppressed, reflexive eye movements.

### Retinal-speed Control

The data from the visual-only and combined conditions of the Main Experiment (Figs. 2 and 3, S1-S6) show a significant (F(3,5) = 28.25, p<0.001) overall effect of heading direction: object discrimination thresholds were consistently greatest for the 0° heading. We hypothesized that this dependence was predominantly due to differences in the self-motion-related component of retinal speed at the object location (*v_s_*) across headings. Specifically, as heading direction is shifted from forward toward lateral, the expected retinal image motion due to self-motion at the location of the object (*v_s_* in Fig. 1B) increases. We therefore repeated the experiment for a subset of subjects while matching optic flow speed at the object location (*v_s_*) across heading directions. This was done by changing the amplitude of self-motion as a function of heading. With the self-motion component of retinal speed (*v_s_*) matched at the location of the object, any remaining effect of heading direction would suggest some dependence of flow-parsing on flow field geometry. In particular, for heading 0, the flow field is radial and there is considerable divergence at the location of the object motion (Fig. 1C). For heading 90, on the other hand, the flow field is laminar and divergence at the location of object motion is minimal (Fig. 1F).

Results from this experiment are illustrated by the green bars in Fig. 2 (S4-S8). When the retinal speed of optic flow at the object location (*v_s_*) was matched across headings, there was no significant influence of heading direction on object discrimination thresholds (F(3,4) = 1.34, p = 0.31). Thus, the overall effect of heading eccentricity on discrimination thresholds in the first experiment appears to result primarily from associated changes in retinal speed. Prior research has demonstrated the dependence of flow parsing on global flow properties [2]. However, given our limited investigation of this question, we did not find evidence that flow-parsing depended on the degree of divergence in the flow field at the location of the object motion.

## Discussion

Estimation of self-motion and object motion are reciprocal parts of the flow-parsing problem, so factors influencing estimation of self-motion may also influence observers’ ability to estimate object motion during self-motion. We examined the influence of vestibular stimulation and heading direction on observers’ ability to discriminate the direction of object motion in the world. Similar manipulations were shown previously to influence heading discrimination [19]–[21], and here we have shown that they also influence object motion discrimination. We found that object discrimination thresholds during self-motion generally decreased when congruent vestibular stimulation accompanied background optic flow, suggesting that vestibular inputs can help parse retinal image motion into self-motion and object motion components.

### Vestibular Facilitation of Optic Flow Parsing

Although the observed effect was small, this is not surprising considering the processes that are likely to be involved. We assume (at least) a two-stage process in which 1) the nervous systems generates a multisensory estimate of self-motion, and 2) uses this estimate to recover object motion in the world by canceling the expected visual consequences of self-motion. Any facilitation due to vestibular stimuli will most likely act by reducing the variability of the multisensory estimate of self-motion described in stage one above. We have studied visual-vestibular heading estimation extensively [17]–[19] and have found that the standard predictions of the Maximum-likelihood Estimation (MLE) model of cue integration are upheld [35]. The predicted improvement in combined heading estimation relative to visual-only is at most ∼√2, and this should occur when visual and vestibular heading estimates are approximately equally reliable.

Over the range of headings investigated here, previous measurements indicate that the reliabilities of visual and vestibular heading estimates vary considerably [21]. For discrimination around a straight forward heading reference, visual heading discrimination thresholds are much more reliable than vestibular thresholds. However, visual heading thresholds increase approximately 5-fold as reference eccentricity increases toward lateral heading directions [Fig. 2B of 21]. Vestibular heading discrimination thresholds also increase with eccentricity of the reference heading, but only approximately 2-fold, for lateral as compared to forward heading directions [Fig. 2A of 21]. Vestibular heading thresholds were never lower than visual thresholds, but were approximately equal for the lateral (90°) heading eccentricity.

Consequently, it is reasonable to expect that vestibular cues are weighted more heavily for eccentric heading directions where their relative reliability is more comparable to that of visual heading cues. By this logic, we expect to see larger vestibular-facilitated decreases in object motion discrimination thresholds for eccentric rather than forward heading directions. Our results are consistent with this hypothesis. Subjects showed little or no improvement in object motion discrimination in the combined condition for forward heading (0°) and the largest improvement for lateral (90°) heading (Fig. 3B). Indeed, the maximum improvement predicted by the MLE model is ∼√2, which is of the same order of magnitude as the largest observed improvements in our experiment (∼20–30%, Fig. 3B).

Note that direct extension of MLE cue integration predictions to our object motion task requires some assumptions. First, the estimate of self-motion should be unbiased, or the bias should remain fairly constant for a given heading direction. Second, the operation that cancels the expected visual consequences of self-motion (described as stage two, above) should introduce little noise into the object motion estimate. If either of these assumptions is substantially violated, the expected improvement in performance in the combined condition will be reduced relative to the MLE-prediction.

While the present results are suggestive, they do not prove conclusively that object motion perception depends directly on heading recovery. Recent work with visual-only stimuli has aimed to test the hypothesis that object motion estimates can be predicted directly from heading estimates in response to an illusory optic flow stimulus [36]. Results of that study are inconsistent with predictions of the strict self-motion-cancellation hypothesis, suggesting that flow parsing does not necessarily depend on heading recovery. Clearly, further research is needed on this topic.

Importantly, an alternative explanation of our results based on a residual TVOR, which might cause a physical (rather than computational) reduction of background optic flow, is inconsistent with our data. Mean eye velocity was small on most trials and was similar for visual-only and combined conditions. We calculated object discrimination thresholds as a function of mean eye velocity and this analysis confirmed that the vestibular facilitation of object discriminability could not be attributed to reflexive eye movements. We suggest instead that vestibular self-motion signals contribute to optic flow parsing computations. Note, however, that a more complete understanding of the role of vestibular signals in flow parsing will require experiments that also measure biases in perceived object motion trajectory due to self-motion. Future studies should examine how vestibular signals modulate the ability of subjects to accurately judge the direction of object motion (relative to the world) in the presence of self-motion.

### Neurophysiological Implications

Given the above considerations, it is striking that we observed an overall decrease in thresholds in the combined condition. Although modest, the improvements in object motion discrimination thresholds that we have observed are likely to be functionally relevant. Moreover, it is possible that the same cortical areas with convergent optic flow and vestibular inputs (e.g., areas MSTd and VIP) [25], [26], [28], [37], [38], which have been implicated in mediating the improvement in heading discrimination thresholds [17]–[19], also mediate improved object motion discrimination during simultaneous vestibular stimulation. Particularly relevant might be a group of cortical multisensory neurons with incongruent visual and vestibular preferences [26], [28], [39]. These cells are sub-optimally stimulated when visual and vestibular signals are congruent, as during self-motion relative to a stationary visual environment in the absence of object motion. On the other hand, they are maximally stimulated by incongruent optic flow and vestibular signals [28], [29], and are therefore ideally suited to signal instances when visual motion does not match the optic flow that might be expected based on vestibular input. This is precisely what occurs during independent object motion. As Wallach proposed [40], the visual system could better estimate object motion during self-motion by ‘canceling’ the effects of self-motion and it is possible that incongruent cells contribute to implementing this cancellation process, such that object motion may be estimated more precisely [41].
